# Network Pharmacology and Experiment Verification-Based Strategy for Exploring the Mechanisms of Shuqing Granule in the Treatment of COVID-19

**DOI:** 10.3390/ph18081216

**Published:** 2025-08-18

**Authors:** Xiaoping Guo, Haoyu Zheng, Yiming An, Yuemeng Song, Tianqi Liu, Zhengjie Zhou, Chuangui Liu, Guoqiang Wang, Fang Wang

**Affiliations:** 1 Department of Pathogen Biology, College of Basic Medical Sciences, Jilin University, Changchun 130021, China; guoxp23@mails.jlu.edu.cn (X.G.); zhenghy22@mails.jlu.edu.cn (H.Z.); anym22@mails.jlu.edu.cn (Y.A.); ymsong23@mails.jlu.edu.cn (Y.S.); liutq9922@mails.jlu.edu.cn (T.L.); zhengjie21@mails.jlu.edu.cn (Z.Z.); 2 National and Local United Engineering R&D Center of Ginseng Innovative Drugs, Changchun 130021, China; liuchuangui@gmail.com

**Keywords:** SARS-CoV-2, ACE2, molecular docking, traditional Chinese medicine

## Abstract

**Background/Objectives:** Coronavirus disease 2019 (COVID-19) has been a global pandemic since 2019, but effective therapeutic treatments for it remain limited. Shuqing Granule (SG) is a traditional Chinese medicine containing ingredients such as indirubin, shinpterocarpin, naringenin, and quercetin. It exhibits anti-inflammatory and antiviral activities as well as broad-spectrum antiviral effects, yet its potential role in the treatment of COVID-19 remains unclear. This study thus aimed to explore the therapeutic effects of SG on COVID-19, with a focus on its potential anti-SARS-CoV-2 activity linked to these bioactive ingredients. **Methods:** The potential therapeutic ability of SG was investigated by combining network pharmacology, molecular docking, and experimental verification. First, key ingredients in SG and their corresponding targets, as well as COVID-19-related targets, were identified. Then, enrichment analyses were performed to highlight potential key pathways. Additionally, molecular docking was conducted to assess the binding capacity of the key ingredients to ACE2. Finally, experiments such as Western blot and ELISA were conducted to verify the effect of SG. **Results:** The results showed that 15 key ingredients such as quercetin in SG could affect overlapping targets such as RELA. Molecular docking results showed that key ingredients in SG, such as isoliquiritigenin, formononetin, shinpterocarpin, indirubin, naringenin, kaempferol, and 7-Methoxy-2-methylisoflavone, might bind to angiotensin-converting enzyme II (ACE2)—a critical receptor in the process of COVID-19 infection—thereby exerting antiviral effects. Experiments such as Western blot and ELISA further demonstrated that SG could reduce inflammation induced by the SARS-CoV-2 S1 protein by 50%. This effect might be achieved by downregulating ACE2 expression by 1.5 times and inhibiting the NF-κB signaling pathway. **Conclusions:** This study confirmed that SG has potential as a candidate for COVID-19 treatment. It also provided a new approach for the application of traditional Chinese medicine in combating the virus.

## 1. Introduction

SARS-CoV-2 is a single-stranded positive-sense RNA virus that is the pathogen of coronavirus disease 2019 (COVID-19), which caused a global pandemic [1,2]. As a public health emergency of international concern, the spread of SARS-CoV-2 has had a catastrophic impact on the global health system and the global economic system [3]. Available evidence suggested that SARS-CoV-2 infection induced immune dysfunction, extensive endothelial damage, complement-associated coagulopathy, and systemic microangiopathy [4]. The most common symptoms in hospitalized patients are fever, dry cough, shortness of breath, fatigue, nausea/vomiting or diarrhea, and myalgia. In addition, complications of COVID-19 include impaired function of the heart, brain, lungs, liver, kidneys, and the coagulation system [5]. Moreover, with the emergence of SARS-CoV-2 variants such as alpha, beta, delta, and omicron, COVID-19 is still spreading widely [6,7]. Humanity faces a daunting task of pandemic prevention, so there is an urgent need to screen for effective therapeutic agents [8,9].

After the outbreak of COVID-19, China actively took effective measures to prevent the pandemic and successfully controlled the domestic epidemic in a short period [10]. It was reported that many traditional Chinese medicine (TCM) prescriptions not only inhibited viral replication directly but also reduced excessive pro-inflammatory responses and tissue damage of the virus [11,12]. TCM has also played an indelible role in treating COVID-19 [13]. Many studies have shown that the combined Chinese and Western medicine treatment group has higher overall efficiency, higher cure rate, lower incidence of serious illness, and shorter hospital stay compared to the group applying only Western medicine methods [14]. A variety of traditional Chinese medicines were also recommended in the Guideline on Diagnosis and Treatment of Coronavirus Disease 2019 (8th version) in China for the treatment of patients with different types of neo-coronavirus pneumonia [15], confirming that TCMs are outstandingly effective in the prevention and treatment of COVID-19. Therefore, it is of great significance to explore the chemical ingredients and target genes of TCM for the treatment of COVID-19, explain its mechanism, and provide more drug candidates.

SG is a kind of Chinese patent medicine containing Isatidis Folium (called Daqingye in Chinese), *Morus alba* L. (Mulberry Leaf), *Rhizoma phragmitis* (called Lugen in Chinese), *Glycyrrhiza glabra* L. (Licorice), and *Gypsum fibrosum* (Gypsum), which has cathartic effects and can detoxify the lungs and stomach and clear heat and toxins. Several clinical studies have confirmed that SG has good efficacy and safety in the treatment of children with acute upper respiratory tract infection (wind-heat and -cold syndrome) [16,17]. Furthermore, SG has also been reported to significantly inhibit the proliferation of EV71 and CoxA16 viruses in vitro [18]. The above results demonstrated that SG may have broad-spectrum antiviral effects, but whether it is effective against COVID-19 remains unclear. Therefore, this study intends to combine network pharmacology, molecular docking, and molecular biology experiments to explore the potential effect of SG against COVID-19.

SARS-CoV-2 has been shown to invade host cells mainly through specific recognition of ACE2 receptors on the cell surface by its Spike protein, which then activates the immune response of antigen-presenting cells (APCs) such as dendritic cells (DCs) [19]. Thus, the expression level of ACE2 is likely to play a key role in the SARS-CoV-2 infection process [20,21]. During the early stages of viral infection, the release of cytokines and chemokines from respiratory epithelial cells, dendritic cells, and macrophages is delayed, leading to a cytokine storm [22]. Several studies have found that increased levels of IL-6 were observed in patients with COVID-19 and that the levels correlate strongly with the severity of the disease [23,24,25,26,27]. Therefore, screening for therapeutic agents that can inhibit ACE2 expression and can resist cytokine/anti-cytokine signaling would benefit the disease prognosis of COVID-19 [28].

In this study, we screened the chemical ingredients and potential targets of SG using network pharmacology. A total of 15 key ingredients such as quercetin were obtained. Then protein–protein interaction (PPI), Gene Ontology (GO), and Kyoto Encyclopedia of Genes and Genomes (KEGG) analyses were performed. In total, 50 core targets and key pathways such as NF-κB, inflammatory bowel disease, and RIG-I-like receptor signaling pathway were obtained. The interactions between ACE2 (PDBID:1r4l) and key ingredients were further performed by molecular docking, among which the docking result of isoliquiritigenin with ACE2 was the most outstanding. In addition, the results of in vitro and in vivo studies further confirmed that SG may exert anti-SARS-CoV-2-induced inflammatory responses by inhibiting ACE2 expression, modulating the NF-κB signaling pathway, and inhibiting IL-6 secretion.

## 2. Results

### 2.1. Obtaining the Chemical Ingredients and Corresponding Targets of SG and COVID-19-Related Genes

**Figure 1:**
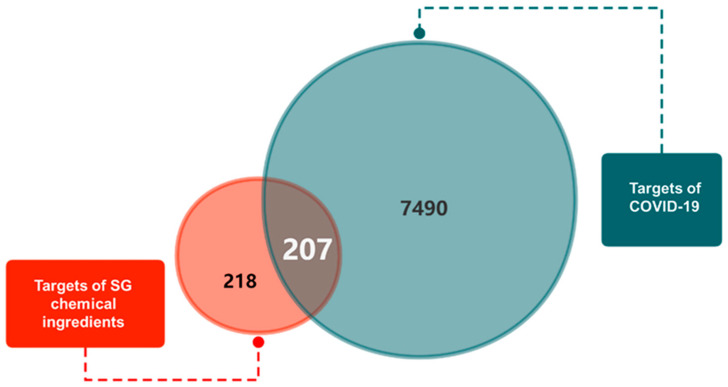
Venn diagram of SG-COVID-19 overlapping targets.

In this study, the chemical ingredients of SG were screened by limiting two indicators, OB and DL. A total of 140 active ingredients were obtained, 95 from Licorice, 34 from Mulberry leaf, 10 from Daqingye, and 1 from Shigao. And 425 targets of the above active ingredients were retrieved. A total of 7697 COVID-19 targets were retrieved. The targets of the SG active ingredients and the targets of COVID-19 were overlapped, and 207 overlapping targets were obtained. The Venn diagram is shown in Figure 1.

### 2.2. Construction of “SG Chemical Ingredient-Overlapping Target-COVID-19” Interaction Network and Identification of Key Ingredients

To explore the potential relationship between the chemical ingredients of SG and COVID-19, a “SG chemical ingredients-overlapping targets-COVID-19” network was constructed (Figure S2), which contained 516 nodes and 4087 edges. In this study, degree centrality was employed as the core topological metric to screen key ingredients. Based on the topological results, 15 chemical ingredients ranking in the top 5% of degree centrality (i.e., degree value ≥ 30) were identified as the key ingredients through which SG may exert therapeutic effects on COVID-19, and these ingredients are listed in Table 1.

### 2.3. Protein–Protein Interaction Network (PPI) Construction and Identification of Core Targets

**Figure 2:**
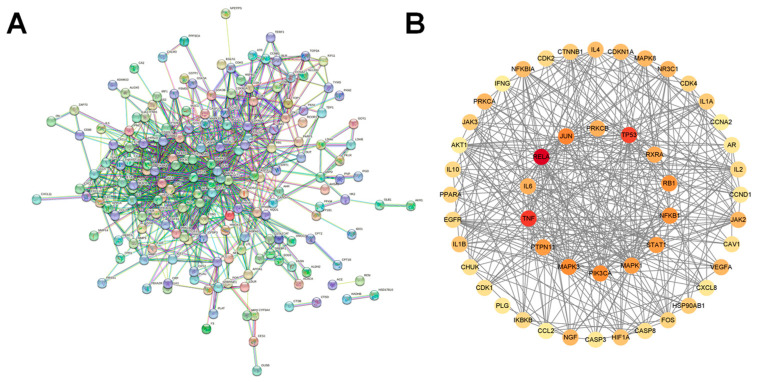
PPI network diagram. (**A**) PPI network exported from String database (**B**) Networks of core targets. Among all core targets, the darker the red, the more important it is.

The 207 overlapping targets were input into the String database, and the PPI network was generated by eliminating each discrete node, as shown in Figure 2A. Each node represented a target, a total of 207 nodes, and the connections between nodes indicated the interactions between targets. The excel data files obtained from the String database analysis were imported into Cytoscape 3.9.0 overlapping target networks for topological analysis, and targets with degree, closeness, and betweenness greater than the corresponding mean values were filtered out as core targets (degree > 9.3708, closeness > 0.0357, betweenness > 315.2359), as shown in Figure 2B. The results suggested that genes such as RELA, TP53, and TNF may play an important role in the treatment of COVID-19.

### 2.4. GO and KEGG Pathway Enrichment Analysis

**Figure 3:**
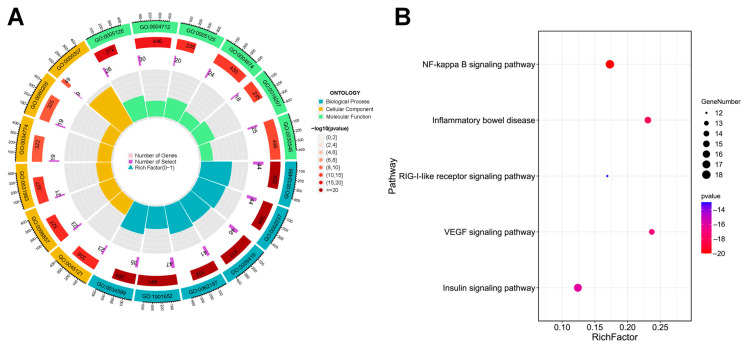
Enrichment analysis results of core targets of SG. (**A**) Top six BP, CC, and MF results in GO annotation analysis. (**B**) Part of KEGG pathway enrichment bubble diagram (*p* < 0.05).

A total of 3144 results were enriched by the GO enrichment method (*p* < 0.01), including 2793 items of BP, 117 items of CC, and 234 items of MF. The top six entries in each section were displayed based on the *p*-value. BP were mainly expressed in response to exogenous infections, inflammation, and oxidative stress. CC were mainly manifested in the inner side of the plasma membrane. MF were mainly expressed in cytokine binding, protein serine/threonine/tyrosine kinase activity, etc., as shown in Figure 3A. The 207 overlapping targets of SG with COVID-19 were analyzed for KEGG pathways using the KEGG online database, and a total of 221 pathways were obtained. Based on the *p*-value and the number of targets enriched in the pathway and in combination with the literature reports, it was concluded that pathways such as NF-κB, inflammatory bowel disease, (and) RIG-I-like receptor signaling pathway may be potential key pathways for SG in the treatment of COVID-19 (Figure 3B) [29,30,31,32,33].

### 2.5. Molecular Docking Results

**Figure 4:**
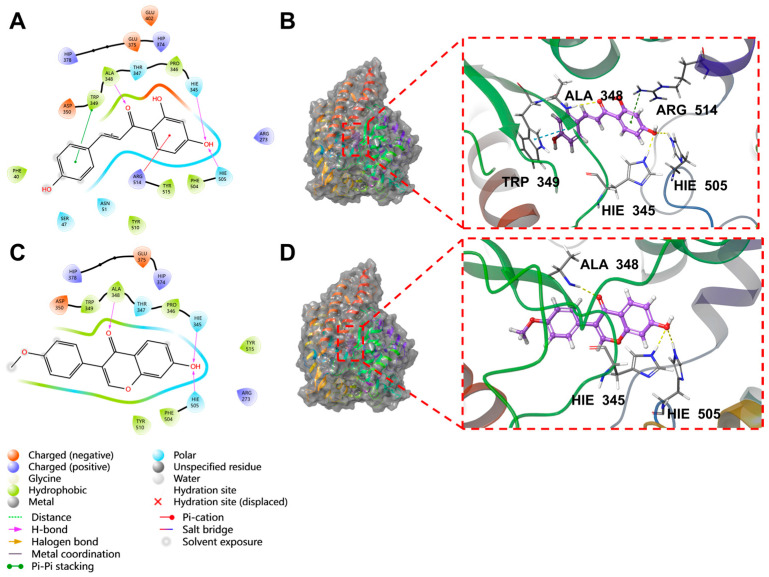
Schematic diagram of interface between ingredients and ACE2 (PDB ID: 1R4L). (**A**) Two-dimensional interaction diagram of isoliquiritigenin and ACE2. (**B**) Molecular dock between isoliquiritigenin and ACE2 crystal structure. (**C**) Two-dimensional interaction diagram of formononetin and ACE2. (**D**) Molecular dock between formononetin and ACE2 crystal structure. Yellow represents hydrogen bonds, blue represents π-π bonds, and green represents π-cation (π cation) bonds.

**Figure 5:**
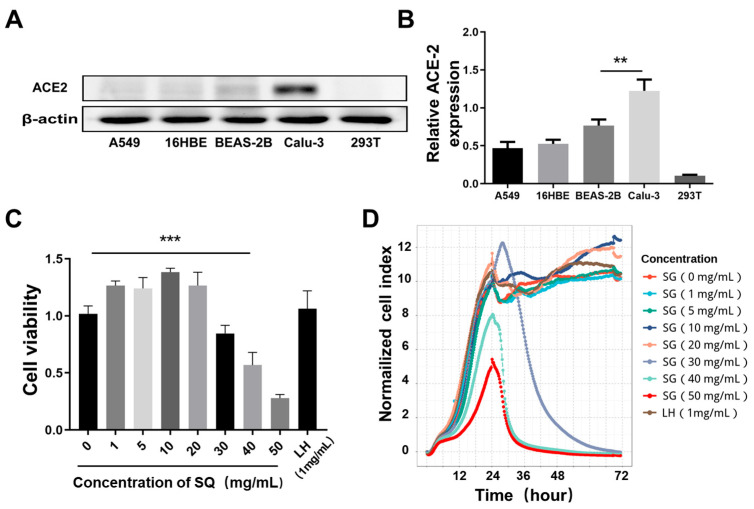
The expression level of ACE2 protein in different cells and the effects of different concentrations of SG on the activity of Calu-3 cells. (**A**) The expression level of ACE2 in A549, 16HBE, BEAS-2B, Calu-3, and 293T cells. (**B**) The statistical analysis of (**A**). (**C**,**D**) The effect of different concentrations of SG and LH (1 mg/mL) on the activity of Calu-3 cells using CCK-8 assay and RTCA assay. Each test was repeated three times. Data were expressed as mean ± SD. ** *p* < 0.01, *** *p* < 0.001.

The docking results of the top eight molecules ranked by XP GScore are shown in Table 2 and Figure 4 and Figures S3–S8. Isoliquiritigenin deeply penetrated into the interior of the ACE2 active pocket, where hydrophobic interactions were formed with the protein residues TRP349, ALA348, PRO346, PHE504, and TYR515. Specifically, the ligand established one hydrogen bond each with residues ALA348, HIE345, and HIE505, a π-π stacking interaction with TRP349, and a π-cation interaction with ARG514 (Figure 5A,B). Formononetin deeply inserted into the interior of the ACE2 active pocket, with hydrophobic interactions formed between the ligand and ACE2 residues including TRP349, ALA348, PRO346, PHE504, and TYR510. Additionally, the ligand formed one hydrogen bond each with residues ALA348, HIE345, and HIE505 (Figure 5C,D). Quercetin deeply penetrated the ACE2 active pocket, with hydrophobic interactions from PRO346, TYR515, PHE274, and LEU370; it formed one hydrogen bond each with GLU406, ASP367, and PRO346, a π-π bond with PHE274, and one π-cation bond each with ARG273 and HIP374 (Figure S3). Kaempferol inserted into the ACE2 active pocket, showing hydrophobic interactions with PHE274, ALA348, PRO346, TYR515, and LEU370; it formed one hydrogen bond each with GLU406 and PRO346, a π-π bond with PHE274, and one π-cation bond each with ARG273 and HIP374 (Figure S4). Indirubin entered the ACE2 active pocket, with hydrophobic interactions from TYR127, LEU144, CYS344, PRO346, and CYS361, and one hydrogen bond with THR371 (Figure S5). Naringenin deeply inserted into the ACE2 active pocket, exhibiting hydrophobic interactions with PHE274, PRO346, TYR515, and LEU370; it formed one hydrogen bond each with PRO346, THR371, and GLU406, and one π-cation bond each with ARG273, HIP374, and ARG518 (Figure S6). Shinpterocarpin penetrated the ACE2 active pocket, with hydrophobic interactions from LEU351, TRP349, ALA348, TYR510, and PRO346, but no non-covalent bonds (Figure S7). 7-Methoxy-2-methylisoflavone deeply entered the ACE2 active pocket, showing hydrophobic interactions with PHE504, PRO346, PHE274, LEU370, and TYR515, and one hydrogen bond each with HIE345, HIE505, and ARG518 (Figure S8).

A comprehensive analysis of XP docking and MM-GBSA results demonstrated that isoliquiritigenin showed the best docking performance with ACE2. Both its docking score and binding free energy were low, indicating that isoliquiritigenin binds sufficiently stably to ACE2. Formononetin, shinpterocarpin, indirubin, naringenin, kaempferol, and 7-Methoxy-2-methylisoflavone exhibit relatively stable binding to ACE2. In contrast, quercetin binds unstably to ACE2.

### 2.6. Screening of Cells with High Expression of ACE2

To investigate whether SG can inhibit the stimulatory effect of SARS-CoV-2 S1 protein on cells, we first screened cell lines with high expression of SARS-CoV-2 Spike protein-binding receptor ACE2. The results are shown in Figure 5A,B; the expression of ACE2 in Calu-3 cells was significantly higher than the other four groups. Therefore, Calu-3 cells were used for subsequent drug toxicity studies. The CCK-8 results indicated that when the SG concentration was greater than 30 mg/mL, the cell’s survival rate decreased significantly (*p* < 0.01), while when the concentration was less than 20 mg/mL, there was no significant difference in the cell survival rate compared with the control group (*p* > 0.05), as shown in Figure 5C. The RTCA technique was also applied to further confirm the non-toxic concentrations of SG acting on Calu-3 cells, as shown in Figure 5D. Similarly to the CCK-8 results, cell viability was not significantly changed when the drug concentration was below 20 mg/mL; however, the number of cells decreased with increasing administration concentrations at SG concentrations of 30, 40, and 50 mg/mL. Furthermore, among the results detected by the two methods, no significant cytotoxicity was observed for LH (1 mg/mL). In summary, 5, 10, and 20 mg/mL were selected as low, medium, and high concentrations for the follow-up experiments.

### 2.7. SARS-CoV-2 S1 Protein Stimulation of Calu-3 Cells Induces Inflammation

**Figure 6:**
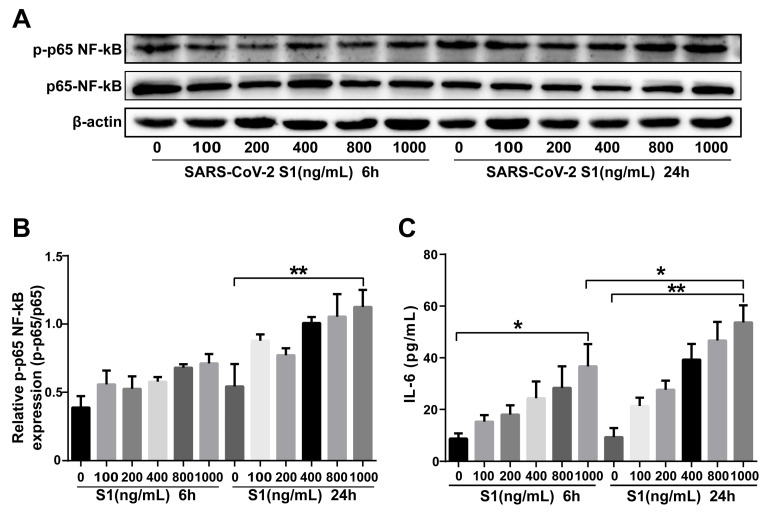
Changes in supernatant IL-6 secretion from Calu-3 cells after SARS-CoV-2 S1 protein stimulation. (**A**,**B**) Levels of total and phosphorylated NF-κB p65 and densitometry at 6 and 12 h post-stimulation by SARS-CoV-2 S1 protein. (**C**) Content of IL-6 in supernatant of Calu-3 cells after SARS-CoV-2 S1 protein stimulation. Each test was repeated three times. Data were expressed as mean ± SD. * *p* < 0.05, ** *p* <0.01.

As shown in Figure 6A,B, the phosphorylation level of NF-κB p65 in each group was increased after SARS-CoV-2 S1 protein stimulation. Compared with the control group, the phosphorylation level of NF-κB p65 increased significantly when the SARS-CoV-2 S1 protein concentration was 1000 ng/mL and the stimulation time was 24 h (*p* < 0.01). The content of IL-6 was also increased with increasing SARS-CoV-2 S1 protein concentration (Figure 6C). The content of IL-6 in the 1000 ng/mL SARS-CoV-2 S1 protein group was significantly increased compared with the control group at the two time points (*p* < 0.05, *p* < 0.01). Compared with the SARS-CoV-2 S1 protein stimulation for 6 h, the content of IL-6 in the 24 h group was increased (*p* < 0.05). These results suggested that 1000 ng/mL SARS-CoV-2 S1 protein stimulation for 24 h was the optimal condition to induce inflammation. In summary, in this study, the SARS-CoV-2 S1 protein concentration was selected as 1000 ng/mL, and the stimulation time was 24 h to construct the cellular inflammation model of SARS-CoV-2 S1 protein-stimulated Calu-3 cells.

### 2.8. SG Inhibited Expression of ACE2 in Calu-3 Cells

**Figure 7:**
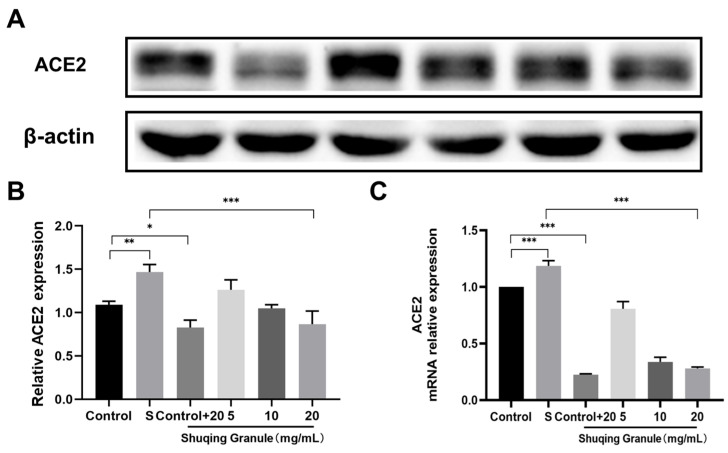
SG inhibited expression of ACE2 in Calu-3 cells. (**A**) Expression levels of ACE2. (**B**) Analysis of band grayscale values of ACE2. (**C**) ACE2 mRNA relative expression. Each test was repeated three times. Data were expressed as mean ± SD. * *p* < 0.05, ** *p* < 0.01, *** *p* < 0.001.

To investigate whether SG had an inhibitory effect on ACE2 expression, we examined ACE2 expression levels before and after SG treatment; the results are shown in Figure 7. Compared with the control group, the ACE2 expression level increased significantly in the SARS-CoV-2 S1 protein stimulation group (S group) (*p* < 0.01) and decreased significantly in the SG alone administration group (control + 20 group) (*p* < 0.05); the ACE2 expression level decreased in a dose-dependent manner with SG concentration in the SARS-CoV-2 S1 protein stimulation with simultaneous administration of SG group (5, 10, 20 group), as shown in Figure 7A,B. We found similar results in ACE2 mRNA relative expression, as shown in Figure 7C, with significantly higher ACE2 mRNA relative expression in the SARS-CoV-2 S1 protein stimulation group compared to the control group (*p* < 0.001), which decreased significantly in the SG alone administration group (control+20) (*p* < 0.0001); the ACE2 mRNA relative expression decreased in a dose-dependent manner with SG concentration in the SARS-CoV-2 S1 protein stimulation with simultaneous administration in the SG group.

### 2.9. SG Inhibited the Phosphorylation Level of NF-κB p65 and the Secretion of IL-6

**Figure 8:**
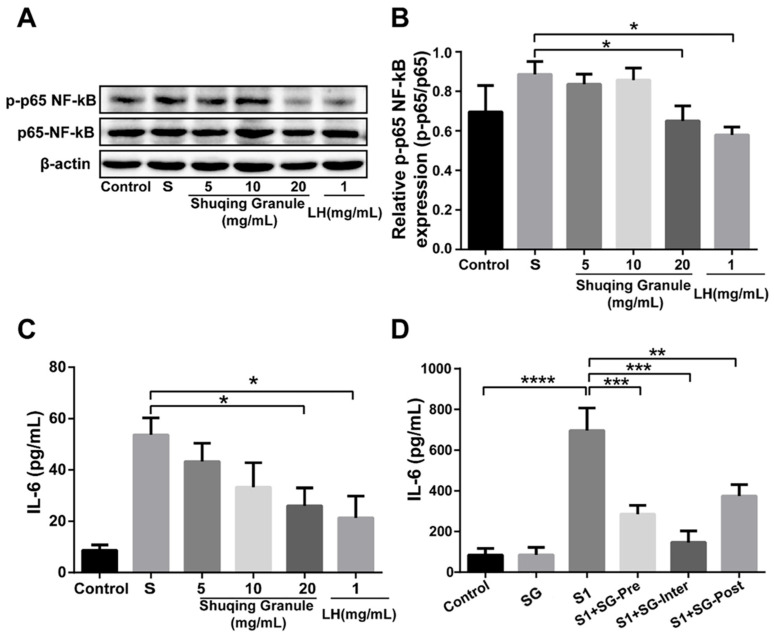
SG inhibited the phosphorylation level of NF-κB p65 and the secretion of IL-6 in Calu-3 cells after stimulation with SARS-CoV-2 S1 protein. (**A**,**B**) The effects of SG on the phosphorylation level of NF-κB p65 and densitometry. (**C**,**D**) The effects of SG on the content of IL-6 in the supernatant of Calu-3 cells and in the serum of mice. Each test was repeated three times. Data were expressed as mean ± SD. * *p* < 0.05, ** *p* < 0.01, *** *p* < 0.001, **** *p* < 0.0001.

To determine whether SG has an inhibitory effect on the inflammatory response induced by SARS-CoV-2 S1 protein, we detected the protein level of NF-κB p65 and the content of IL-6 after SG treatment, with Lianhuaqingwen capsule as the positive drug (LH group). The results are shown in Figure 8A,B. Compared with the SARS-CoV-2 S1 protein stimulation group, the phosphorylation level of NF-κB p65 was significantly decreased in the high concentration of SG (20 mg/mL), and the results were similar to those in the LH group (*p* < 0.05). However, there was no significant change in the SG (5 and 10 mg/mL) groups (*p* > 0.05).

The content of IL-6 in Calu-3 cells was significantly increased after SARS-CoV-2 S1 protein stimulation (*p* < 0.05), and after SG intervention treatment, the content of IL-6 in the SG (20 mg/mL) group was significantly decreased (*p* < 0.05), as shown in Figure 8C.

In addition, we further constructed an animal model stimulated by SARS-CoV-2 S1 protein to evaluate the inhibitory effect of SG on IL-6, as shown in Figure 8D. Compared with the control group, the content of IL-6 was significantly increased in the model mice (*p* < 0.05). Compared with the S1 group, the content of IL-6 in the serum of the mice in the SG treatment group was significantly decreased (*p* < 0.01, *p* < 0.001, *p* < 0.0001). The above results suggested that SG can effectively inhibit the inflammatory response triggered by SARS-CoV-2 S1 protein stimulation.

## 3. Discussion

The outbreak of the COVID-19 pandemic caused by SARS-CoV-2 infections in late 2019 has wreaked havoc on people across the world [3]. Despite the current high SARS-CoV-2 vaccine coverage, some immunocompromised populations are at increased risk of SARS-CoV-2 infection and serious illness, especially older adults who live in concentrated populations [34]. So, drug development targeting the treatment of COVID-19 is still needed [35,36]. In TCM, COVID-19 has been classified as an “epidemic disease.” The reason is that exogenous pathogens and toxins are sensed by the human body. The pathogenesis is mainly “wet, heat, poison, stasis, deficiency” and the lesions are mainly in the spleen, lungs, and stomach [37]. During the SARS outbreak, TCM made a significant contribution as an auxiliary means [38]. It has been demonstrated that in the treatment of COVID-19, TCM has also played an indispensable role. Active ingredients such as quercetin, puerarin, porphyrin, quercetin-7-O-β-D-glucoside, and patchouli alcohol in Patchouli have good bindings to SARS-CoV-2’s main protease (Mpro) and can reduce viral replication [39]. The seven active ingredients in rhubarb, including β-sitosterol and aloe rhodopsin, have multi-pathways and multi-targeted effects, including antiviral, anti-inflammatory, anti-oxidative stress, anti-apoptosis, and modulation of the body’s immune system [40]. In addition, the active ingredients of TCM, such as glycyrrhizin and baicalin, have inhibitory effects on viral activity [41,42]. It has been reported that in most of the mildly ill patients treated with TCM, the time for the disappearance of clinical symptoms and recovery of body temperature was shortened. The average hospitalization time and the incidence of severe illness were significantly reduced, and the improvement rate of CT images increased [43].

Network pharmacology is a part of bioinformatics, which integrates bioinformatics and systems medicine to provide a new approach to gain a systematic understanding of traditional complex herbal formulations and to understand the role of TCM on diseases, and has been widely used in TCM research and advanced drug discovery in recent years [44,45,46,47]. Molecular docking techniques, which can spatially dock small molecules to large molecules and score how well they bind, are now commonly used in molecular structure-based drug design studies [48]. In this study, according to the results of network pharmacology and molecular docking, it was found that the key ingredients with higher comprehensive scores in SG included shinpterocarpin, naringenin, quercetin, indirubin, kaempferol, etc. We found that these ingredients had a high binding ability to ACE2. In other studies, quercetin and kaempferol have also been shown to bind well to the 3-Cymotrypsin-like protease (3CL pro) of SARS-CoV-2, with therapeutic potential for COVID-19, which is consistent with our findings [49,50,51]. Indirubin has been shown to exert anti-inflammatory effects by inhibiting the NF-κB and MAPK signaling pathways [52]. Naringenin, an anti-inflammatory and antiviral flavanone, is considered a potential anti-COVID-19 ingredient [53]. Therefore, various ingredients in SG may have therapeutic effects on COVID-19.

The invasion of SARS-CoV-2 into host cells is mediated by the transmembrane Spike glycoprotein, of which the S1 protein is responsible for binding to the host cell ACE2 receptor; the S2 protein mediates the fusion of the virus and the cell membrane [54]. It has been shown that SARS-CoV-2 can upregulate the expression level of ACE2 to promote its infection and transmission, and the inhibition of ACE2 expression by drugs may be the needed direction for drug development [55,56]. Therefore, we first examined the effect of SG on ACE2 expression level and found that SG significantly reduced ACE2 expression levels, and thus we hypothesized that SG might reduce SARS-CoV-2 infection by decreasing ACE2 expression and thus SARS-CoV-2 infection.

Studies have shown that SARS-CoV-2 S1 protein can stimulate the occurrence of inflammatory responses [57,58]. Thus, we used recombinant SARS-CoV-2 S1 protein to construct an inflammation model in vitro and in vivo. The results showed that SARS-CoV-2 S1 protein stimulation can promote the phosphorylation of NF-κB and promote the secretion of IL-6. SG treatment can effectively inhibit the phosphorylation level of NF-κB and inhibit the secretion of IL-6. IL-6 secretion levels were higher in COVID-19 deaths than in survivors, suggesting that COVID-19 mortality may be due to a virus-activated cytokine storm including IL-6 [59,60,61,62,63,64]. The NF-κB signaling pathway plays an important role in the inflammatory response and immune response by regulating the gene expression of factors associated with inflammatory cell infiltration [65]. It has been shown that the emergence of cytokine storms associated with disease deterioration in COVID-19 patients is mainly derived from inflammatory responses driven by NF-κB [4,60]. In summary, we speculate that SG can regulate the NF-κB signaling pathway, inhibit the release of pro-inflammatory factors such as IL-6, and slow down the occurrence of a cytokine storm, thereby exerting a therapeutic effect on COVID-19. The findings of this study provide a basis for further investigation of SG as a potential therapeutic strategy for COVID-19. However, it must be noted that the present study still has some limitations. First, bioinformatics techniques such as network pharmacology rely on data within existing databases, which inevitably have certain errors due to the different sources of original experimental data from existing databases. Therefore, we need to combine more literature mining and experimental evidence in the future to discover more potential chemical ingredients. Second, it is important to acknowledge that stimulation by the SARS-CoV-2 S1 protein, while serving as a valuable experimental model to explore specific pathogenic mechanisms, can only partially simulate the inflammatory response caused by actual SARS-CoV-2 infection. This is because the S1 protein, as a single structural component of the virus, primarily mediates key processes such as receptor binding (e.g., interaction with ACE2) and initial activation of certain signaling pathways, but it cannot replicate the full range of biological events triggered by a live viral infection. In response to these shortcomings, the focus of future work will be on conducting in vivo and in vitro experiments of SARS-CoV-2 infection, further validating the effects of SG on SARS-CoV-2 infection. These studies will provide a scientific basis for the use of TCM in the prevention and treatment of COVID-19.

## 4. Materials and Methods

### 4.1. Obtaining the Chemical Ingredients and Corresponding Targets of SG and COVID-19-Related Genes

First, we searched the main chemical ingredients of SG in the Traditional Chinese Medicine Systems Pharmacology (TCMSP) database (https://www.tcmsp-e.com/load_intro.php?id=43, accessed on 11 December 2021) and Symmap database (http://www.symmap.org/, accessed on 11 December 2021). Drug-likeness (DL) ≥ 0.18 and Oral Bioavailability (OB) ≥ 30% were set as the screening criteria to obtain the chemical ingredients meeting the requirements. The screened chemical ingredients were uploaded to TCMSP (https://www.tcmsp-e.com/load_intro.php?id=43, accessed on 11 December 2021), Symmap (http://www.symmap.org/, accessed on 11 December 2021), and SwissTarget Prediction (http://www.swisstargetprediction.ch/, accessed on 11 December 2021) databases to query and screen the target information. The screened target names were entered into the Uniprot (https://sparql.uniprot.org/ accessed on 11 December 2021) database to obtain standardized gene abbreviations, which were de-duplicated and used for subsequent studies. COVID-19-related target genes were obtained through the GeneCards (https://www.genecards.org/, accessed on 11 December 2021), Therapeutic Target Database (https://db.idrblab.net/ttd/, accessed on 11 December 2021), and MalaCards (https://www.malacards.org, accessed on 11 December 2021) databases. The drug chemical ingredient targets were mapped to disease-related targets to obtain overlapping targets as potential targets for SG to treat COVID-19 and to map the Venn diagram.

### 4.2. Construction of “Chemical Ingredient-Overlapping Target-COVID-19” Interaction Network and Identification of Key Ingredients

SG chemical ingredients, COVID-19, and overlapping targets were data organized to generate data files (data.xlsx) and type files (type.xlsx). The targets were categorized according to the classification of cytokine, receptor, protein, transporter, kinase, enzyme, and others. The data were imported into Cytoscape 3.9.0 software, and the interaction network of “chemical ingredient-overlapping target-COVID-19” was established. The network was topologically characterized, and the chemical ingredients with the top 5% values were screened as the key ingredients of SG in the treatment of COVID-19.

### 4.3. Construction of Protein–Protein Interaction Network (PPI) and Identification of Core Targets

PPI networks were constructed using the STRING database version 11.5 (released 12 August 2021–26 July 2023; available at https://string-db.org/, accessed on 11 December 2021) for the species Homo sapiens (taxid: 9606). The intersecting targets were entered into the database, and the confidence interval was set to 0.90 to export the protein interaction network diagram and related information. The data were imported into Cytoscape 3.9.0, the network was topologically analyzed to obtain the relevant topological parameters, and the mean values were filtered to be greater than degree, closeness, and betweenness as the core targets, respectively.

### 4.4. Enrichment Analysis

The overlapping targets were uploaded to the Metascape (https://metascape.org/, accessed on 11 December 2021) database for GO Biological Process (BP), Cellular Component (CC), and Molecular Function (MF) analysis. In addition, the overlapping targets were imported into the Metascape database to obtain signaling pathways that might be related to the overlapping targets, and the pathways closely related to COVID-19 were screened out from all the enriched pathways by combining with the literature as the key pathways in this study. The screened pathways were imported into the Omicshare cloud platform for KEGG enrichment analysis, in which the threshold was set at *p* < 0.05, resulting in a KEGG bubble map.

### 4.5. Molecular Docking

It was found that the key to the infection of human cells by SARS-CoV-2 is the binding of the Spike protein to the human ACE2 protein, allowing the virus to invade the body and cause disease [66]. The crystal structure of ACE2 (PDB ID: 1R4L) was retrieved from the RCSB PDB database and preprocessed using Schrödinger’s Protein Preparation Wizard, involving protein preprocessing, the regeneration of native ligand states, the optimization of hydrogen bond assignments, energy minimization, and water removal. The 2D SDF files of eight ingredients were processed via Schrödinger’s LigPrep to generate all 3D chiral conformations. The active site of ACE2 was identified using the ReceptorGrid Generation module, with an optimal enclosing box encapsulating the ligand in the 1R4L crystal. High-precision XP docking was performed between the preprocessed ligands and ACE2’s active site, where lower scores indicated lower binding free energy and higher stability. MM-GBSA analysis was further conducted, with MM-GBSA dG Bind approximating the binding free energy and lower values reflecting higher binding stability. This method has been reported in many studies and has good reliability [67].

### 4.6. Drugs, Cells, and Animals

SG was purchased from Jilin Huakang Pharmaceutical Co. (Lot Number: 201205000, Dunhua, China). UPLC-QTOF-MSE was applied to identify 18 common peaks in SG (Figure S1; Tables S1 and S2). Lianhuaqingwen capsule was obtained from Yiling Pharmaceutical Co. (Lot Number: A2008137, Shijiazhuang, China). SARS-CoV-2 S1 protein (Catalog Number: 40591-V08B1) was purchased from Sino Biological (Beijing, China).

All cell lines were provided by the Department of Pathogenic Biology, Basic Medical College, Jilin University. A549, 16HBE, 293T, and BEAS-2B cells were cultured in Dulbecco’s Modified Eagle’s medium (DMEM; Catalog Number: C11965500BT, Gibco, GrandIsland, USA). Calu-3 cells were cultured in Minimum Essential Medium (MEM; Catalog Number: 41500, Solarbio, Beijing, China). All measurements were performed in triplicate.

Additionally, 8-week-old Kunming mice (SYXK(Ji)2019-0015), half male and half female, were used in this study. All mice were raised under the same conditions with standard laboratory food and water (temperature: 21–23 °C; relative humidity: 40–60%; 12 h light/dark cycle with light on at 7.00 a.m.) and housed in a Specific Pathogen Free (SPF) environment. The protocol of animal experiments was approved by the Institutional Animal Care and Use Committee of Jilin University (permit No. 2021-0035).

### 4.7. Cytotoxicity Assay

Cytotoxicity was measured using the CCK-8 kit (Catalog Number: CK04, Dojindo, Kyushu Island, Japan) and Real Time Cellular Analysis (RTCA, ACEA, Jiangsu, China). Calu-3 cells in 96-well plates were incubated with different concentrations of SG (0, 1, 5, 10, 20, 30, 40, 50 mg/mL) and LH (1 mg/mL, positive control). After 24 h, the cells were stained with CCK-8 solution for 2 h. Then the absorbance at 450 nm was determined. Calu-3 cells (approximately 10,000 cells per well) were seeded into the RTCA-specific 16-well plates for 24 h, and then different concentrations of SG were added to each well. The RTCA software (RTCA software Lite) displayed the growth index of the cells according to the live cell resistance value.

### 4.8. Murine Model Establishment

**Figure 9:**
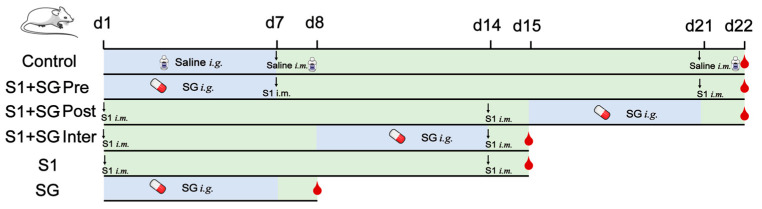
A schematic diagram of the establishment of the in vivo model and the treatment. The dosage of SARS-CoV-2 S1 protein was 50 μg/kg. The dosage of SG was 4.68 mg/kg. *i.g.*: intragastric administration; *i.m.*: intramuscular injection. Blood drops represented the sampling of mice.

Mice were randomly divided into five groups with 6 mice in each group: control group, SG treatment group (SG Group), S1 immunization group (S1 Group), pre-immunization + SG (S1 + SG-Pre), inter-immunization + SG (S1 + SG-Inter), post-immunization + SG (S1 + SG-Post). The control group received intragastric administration of saline from d1 to d7, intramuscular injection of normal saline on d7 and d21, and blood collection on d22. The S1 + SG-Pre group received SG intragastric administration from d1 to d7, intramuscular injection of SARS-CoV-2 S1 protein was performed on d7 and d21, and blood collection was conducted on d22. The S1 + SG-Post group received intramuscular injection of SARS-CoV-2 S1 protein on d1 and d14, SG intragastric administration from the d15 to d21, and blood collection on d22. The S1 + SG-Inter group received intramuscular injection of SARS-CoV-2 S1 protein on d1 and d14, SG intragastric administration from d8 to d14, and blood collection on d15. The S1 group received intramuscular injection of SARS-CoV-2 S1 protein on d1 and d14, and blood was collected on d15. The SG group received SG intragastric administration from d1 to d7, and blood collection was conducted on d8. The experimental outline is shown in Figure 9. The immunization dose of SARS-CoV-2 S1 protein was 50 μg/kg, and the volume was 100 μL (S1: aluminum adjuvant = 3:1) intramuscular injection (*i.m*) per mouse. SG was dissolved in distilled water and intragastrical administration (*i.g*) at a dose of 4.68 mg/kg. Mouse anesthesia was performed using airway inhalation of isoflurane.

### 4.9. Enzyme-Linked Immunosorbent Assays (ELISAs)

Human and mouse IL-6 ELISA kits were purchased from R&D Systems (Catalog Number: D6050B, M6000B-1, Minneapolis, MN, USA). Cell culture supernatants and mouse sera from each group were collected and the content of IL-6 was measured. All measurements were performed in triplicate.

### 4.10. RT-PCR Analysis

Total RNA from Calu-3 cells was extracted and then the concentration and purity were measured using a microplate reader. Reverse transcription was performed using total RNA with the ReverAid First Strand cDNA Synthesis Kit (Catalog Number: K1621, Thermo Scientific, Waltham, MA, USA). cDNA was amplified with specific primers predesigned from Shenggong (Shanghai, China). Real-time PCR was performed using the following conditions: 95 °C for 10 min and 40 cycles of 95 °C for 15 s, 60 °C for 30 s, and 95 °C for 15 s, and a final dissolution curve from 60 °C to 95 °C, with a 0.3 °C increase per second [68]. The sequence of PCR primers is as follows: ACE2, sense: TGTACCTGTTCCGATCATCTGTTGC, and antisense: TAGCCACTCGCACATC CTCCTC. The human-derived GAPDH primers were purchased from Shenggong (Shanghai, China). A relative quantification method was used to calculate the difference in RNA expression.

### 4.11. Western Blot

The total proteins of the samples were extracted from the cells and homogenized in RIPA lysis buffer (Catalog Number: R0020, Solarbio Technology, Beijing, China). The protein concentrations of the samples were detected using the BCA kit (Catalog Number: P0012, Beyotime, Shanghai, China). Protein samples were separated by SDS-PAGE gel electrophoresis (10% glycine-based gels) and transferred to polyvinylidene difluoride membranes (Catalog Number: IPVH00010, Millipore Corp, Bedford, MA, USA). The membranes were then blocked with 5% (*w*/*v*) BSA (Catalog Number: ST023, Beyotime, Shanghai, China) for 2 h and incubated overnight at 4 °C with primary antibodies (antibodies against β-actin, ACE2, P65, and p-P65). After washing in Tris-Buffer saline early the next morning, the membranes were incubated with secondary horseradish peroxidase (HRP)-labeled antibodies for 1 h. Finally, enhanced chemiluminescence (Catalog Number: P0018S, Beyotime, Shanghai, China) was applied to the membranes for signal detection. The intensity of each stripe was quantified using ImageJ software V1.8.0.112 (National Institutes of Health, Bethesda, MD, USA). Antibodies against β-actin, ACE2, P65-NF-κB, and p-P65-NF-κB were purchased from Abcam (UK).

### 4.12. Statistical Analysis

All the analyses were performed in GraphPad Prism (version 6; GraphPad Software, San Diego, CA, USA). Data were presented as the mean ± standard deviation (SD). Differences in multiple groups were determined by one-way ANOVA with Tukey’s honest significant difference (HSD) test. *p*-value < 0.05 was considered statistically significant.

## 5. Conclusions

This study explored the potential therapeutic effect of SG on COVID-19 by combining network pharmacology, molecular docking, and experimental verification, and investigated its possible mechanism. Network pharmacology suggested that the therapeutic effect of SG on COVID-19 may be related to 15 key ingredients such as quercetin and isoliquiritigenin, core targets such as RELA, TP53 and TNF, and signaling pathways such as NF-κB. The molecular docking results suggested that isoliquiritigenin was the key ingredient most likely to bind to ACE2. In addition, we preliminarily demonstrated that SG inhibited ACE2 expression and attenuated SARS-CoV-2 S1 protein-induced inflammatory response by downregulating the activation level of the NF-кB signaling pathway and inhibiting IL-6 secretion. These results suggested that SG may be an effective drug for the treatment of COVID-19. This study provided a new idea for the treatment of COVID-19 with TCM, which could be explored using SARS-CoV-2 in future research to better mimic the inflammatory response after viral infection, and also provided a new perspective for the secondary development of TCM.

## Figures and Tables

**Table 1:** Key ingredients of SG in treatment of COVID-19.

No.	Chemical Ingredients	Degree	Source	Chemical Structure
1	Quercetin	726	Mulberry leaf, Lugen	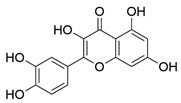
2	Kaempferol	231	Mulberry leaf, Lugen	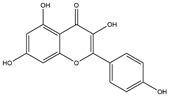
3	Acetic Acid	109	Mulberry leaf	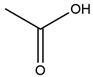
4	Aceton	61	Mulberry leaf	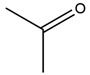
5	Eugenol	57	Mulberry leaf	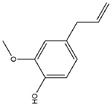
6	Naringenin	55	Licorice	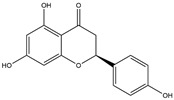
7	Calcium sulfate	52	Shigao	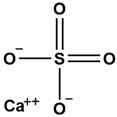
8	Salicylic Acid	47	Daqingye	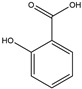
9	Formononetin	40	Licorice	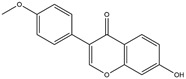
10	Indirubin	35	Daqingye	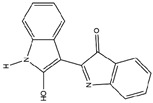
11	7-Methoxy-2-methyl isoflavone	34	Licorice	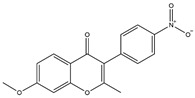
12	Isoliquiritigenin	34	Licorice	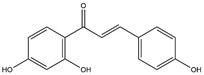
13	Anethole	33	Mulberry leaf	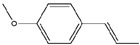
14	Dbp	32	Mulberry leaf	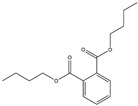
15	Shinpterocarpin	30	Licorice	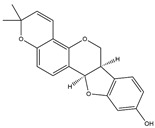

**Table 2:** Molecular docking score of key ingredients in SG with ACE2 (PDB ID: 1R4L).

No.	Ingredient	XP GScore	MMGBSA dG Bind
1	Isoliquiritigenin	−6.005	−44.40
2	Formononetin	−5.958	−40.39
3	Quercetin	−5.892	−29.34
4	Kaempferol	−5.161	−30.97
5	Indirubin	−4.900	−32.40
6	Naringenin	−4.471	−31.22
7	Shinpterocarpin	−4.337	−35.88
8	7-Methoxy-2-methylisoflavone	−2.252	−30.89

## Data Availability

The authors confirm that the data supporting the findings of this study are available within the article.

## Supplementary Materials

The following supporting information can be downloaded at: https://www.mdpi.com/article/10.3390/ph18081216/s1. Figure S1: Base peak intensity chromatograms of SG in ESI+ and ESI- modes. Table S1: The chemical ingredients identified from SG [69,70,71,72,73,74,75,76,77,78,79,80]. Table S2: The structures and names of chemical ingredients identified from SG. Figure S2: Chemical ingredients of SQ–overlapping targets–COVID-19. Figure S3: Schematic diagram of interface between quercetin and ACE2 (PDB ID: 1R4L). Figure S4: Schematic diagram of interface between kaempferol and ACE2 (PDB ID: 1R4L). Figure S5: Schematic diagram of interface between indirubin and ACE2 (PDB ID: 1R4L). Figure S6: Schematic diagram of interface between naringenin and ACE2 (PDB ID: 1R4L). Figure S7: Schematic diagram of interface between shinpterocarpin and ACE2 (PDB ID: 1R4L). Figure S8: Schematic diagram of interface between 7-Methoxy-2-methylisoflavone and ACE2 (PDB ID: 1R4L).
