# Supplementary material for: Network Pharmacology and Experiment Verification-Based Strategy for Exploring the Mechanisms of Shuqing Granule in the Treatment of COVID-19
Source: Pharmaceuticals (Basel). 2025 Aug 18;18(8):1216. doi: 10.3390/ph18081216 (PMC12389595; doi:10.3390/ph18081216)
Supplement: Supplementary file 1 [file pharmaceuticals-18-01216-s001.zip › pharmaceuticals-3780339-supplementary.pdf]

Drugs and Reagents

SG was purchased from Jilin Huakang Pharmaceutical Co., (Lot Number: 201205000). Methanol, acetonitrile (Fisher Scientific International Inc.); Formic acid was MS grade (Sigma-Aldrich); Sodium formate (Waters Technologies, Inc.); Leucine-enkephalin (Waters Technologies, Inc.); The water used for mass spectrometry was Watson's purified water (Guangzhou Watson's Food & Beverage Co., Ltd.); other reagents were analytically pure.

Sample Preparation

1.0 g of SG was added to 70 mL of methanol and then extracted at reflux for 3 times, filtered, and the filtrate was mixed, concentrated under reduced pressure, dried, and fixed to 10 ml by addition of methanol. Finally, passed through 0.22 μm membrane, and left to be used.

UPLC-QTOF-MS

UPLC-QTOF-MSE analysis was performed on a Waters Xevo G2-XS QTOF mass spectrometer (Waters Co., Milford, MA, USA) equipped with a UPLC system through an electrospray ionization (ESI) interface. An ACQUITY UPLC BEH C18 (100 mm × 2.1 mm, 1.7 μm) from Waters Corporation (Milford, MA, USA) was used for the chromatographic separation. The mobile phases were composed of eluent A (0.1% formic acid in water, v/v) and eluent B (0.1% formic acid in acetonitrile, v/v) with flow rate of 0.35 mL/min. The elution conditions applied were: 0→2 min, 10% B; 2→26 min, 10~90% B; 26→28 min, 90% B; 28→29, 90~10% B; 29→35 min, 10% B. The temperature of the autosampler and the UPLC column manager were set at 15 ℃ and 30 ℃ respectively. The weak wash solvent and strong wash solvent were mixtures of 90/10 and 10/90 water/acetonitrile, respectively. In MSE mode, the mass spectrum was recorded between 100 and 1500 Da. The positive mode conditions were: cone voltage, 40 V; capillary voltage, 2.6 kV; desolvation temperature, 400 ℃; source temperature, 150 ℃; cone gas flow, 50 L/h; desolvation gas flow, 800 L/h. Negative mode conditions were identical to the positive mode conditions except for capillary voltage (2.2 kV). In MSE mode, data acquisition was performed via the mass spectrometer by rapidly switching from a low-collision energy (CE) scan to a high-CE scan during a single LC run. The low-energy and high-energy channel energies were set to 6 V and 20-40 V, respectively, and the real-time correction fluid was

Leucine enkephalin (m/z 556.2771 in positive mode and 554.2615 in negative mode)

Data Analysis

First, the chemical structure of the SG was introduced into the analytical method by reviewing the literature and the data was compressed on the Masslynx™ workstation using the Waters Compression Archival Tool 1.10. The UNIFI software was then used to screen and identify the data, calculate molecular formulae and analyze fragment breaks. Next, a screening process was established to set the mass error to ±5 ppm with a response value over 3000; Finally, the Retention time, Formula, and Calculated mass of the chemical components were identified by searching the mass spectrometry databases such as PubMed, ChemSpider, Mass Bank, and METLIN, and by referring to the relevant literature.

Result

It was characterized using UNIFI and MakerLynx XS V4.1 software (Waters, Milford, CT, USA), and the base-peak ion flow chromatograms in positive and negative ion modes are shown in Figure S1. By comparing with the control or analyzing the exact molecular weight and typical fragments, 18 chemical ingredients were identified in SG in this study as shown in Tables S1 and S2. However, as shown in the base-peak ion flow chromatogram, there are still many peaks with good response values to be further analyzed and identified.

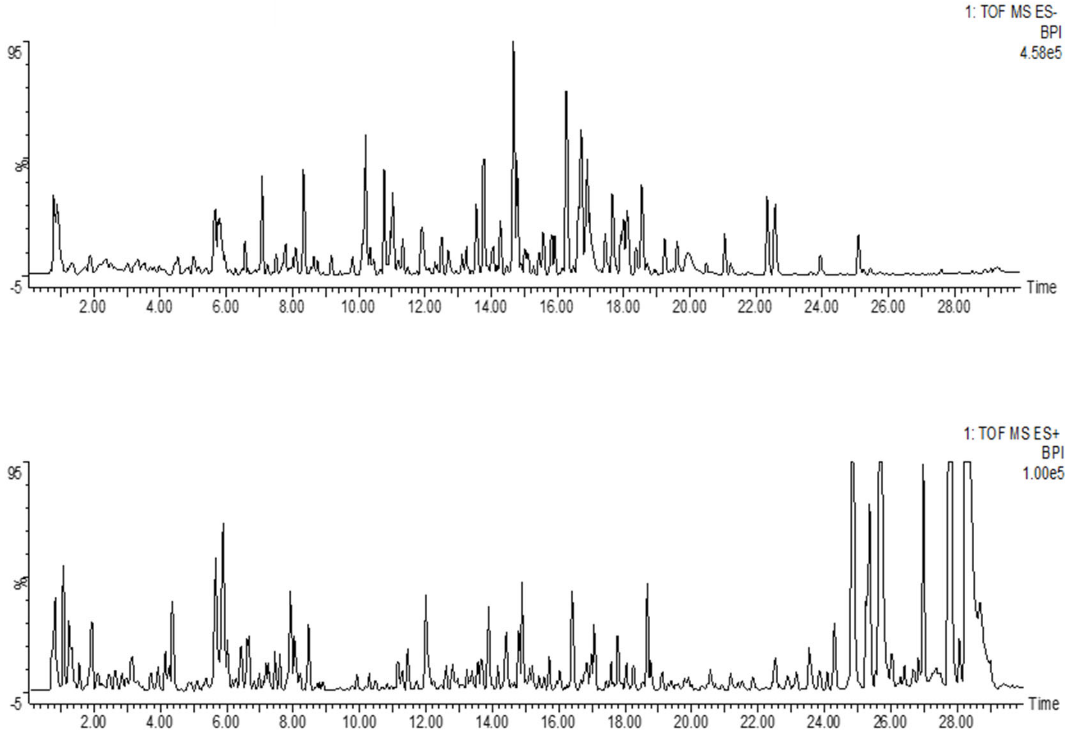

Figure S1. Base peak intensity chromatograms of SG in ESI+ and ESI- modes

Table S1. The chemical ingredients identified from SG

No.	tR (min)	Formula	Calculated mass (Da)	Theoretical mass (Da)	Mass error (ppm)	MSE fragmentation	Identification	Ref.
1	3.15	C27H30O15	594.1560	594.1585	1.5	595.1686[M+H]+ 577.1568[M+H-OH]+ 433.1038[M+H-Rha]+	Rutin	[69]
2	4.16	C27H30O15	594.1585	594.1585	0.0	595.1585[M+H]+ 433.1136[M+H-Glu]+ 271.0613[M+H-2Glu]+	Vicenin-2	[70]
3	5.66	C21H20O10	432.1052	432.1056	-0.5	431.0980[M-H]- 269.0458[M-H-Glu]- 161.0476[M-H-Glu-C6H5O]-	Apigenin 7-O-β-D-glucopyranoside	[70]
4	5.77	C26H30O13	550.1682	550.1684	-0.5	549.1615[M-H]- 399.1118[M-H-Api]- 255.0677[M-H-Api-Glu]-	Liquiritin apioside	[71]
5	5.89	C15H12O4	256.0754	256.0736	1.9	257.0824[M+H]+ 239.0758[M+H-OH]+ 163.0416[M+H-C6H5O]+ 137.0264[M+H-C6H5O-C2H2]+	Isoliquiritigenin	[71]
6	5.93	C15H10O7	302.0443	302.0427	1.7	303.0523[M+H]+ 285.0397[M+H-H2O]+ 109.0323[M+H-C9H6O5]+	Quercetin	[72]
7	7.05	C7H6O3	138.0329	138.0317	1.2	137.0254[M-H]- 93.0367[M-H-COOH]-	Salicylic acid	[69]
8	9.81	C15H10O5	270.0454	270.0526	-0.2	269.0467[M-H]- 161.0227[M-H-OH-C6H5O]- 151.0048[M-H-C6H5O-C2H2]- 117.0326[M-H-C7H5O4]-	Apigenin	[73]
9	11.34	C16H12O4	268.0775	268.0736	2.0	267.0674[M-H]- 252.0428[M-H-CH3]- 223.0414[M-H-C2H5O]-	Formononetin	[74]
10	12.01	C42H62O16	822.4029	822.4038	-0.9	823.4090[M+H]+ 647.3773[M+H-C6H8O6]+ 471.3469[M+H-C12H16O12]+ 453.3357[M+H-C12H16O12-H2O]+	Glycyrrhizic acid	[71]
11	12.01	C28H46O3	430.3476	430.3447	2.9	453.3357[M+Na]+ 435.3248[M+Na-H2O]+ 417.3258[M+Na-2H2O]+ 357.2421[M+Na-C5H11]+	Ergosta-6,22-diene-3β,5α,8α-triol	[75]

12	13.78	C21H20O6	368.1259	368.1260	-0.1	367.1181[M-H]-<br>335.01701[M-H-OCH3]-<br>309.0340[M-H-CH3-C3H7]-	Glycycoumarin	[76]
13	14.06	C22H26O7	402.1679	402.1679	0.1	401.1607[M-H]-<br>369.0985[M-H-2CH3]-<br>355.1566[M-H-COO]-<br>325.1084[M-H-CH2COO-CH3]-<br>219.0672[M-H-CH2-C6H5O3]-	Traxillagenin	[77]
14	14.81	C20H18O6	354.1125	354.1103	2.2	355.1221[M+H]+<br>337.1094[M+H-OH]+<br>299.0574[M+H-C4H8]+<br>179.0358[M+H-C6H5O2-C5H9]+	Gancaonin L	[71]
15	14.91	C21H22O4	338.1537	338.1512	1.9	339.1603[M+H]+<br>307.1329[M+H-OCH3]+<br>271.1000[M+H-C5H8]+<br>245.1172[M+H-C6H5O]+	Licochalcone A	[78]
16	15.10	C21H18O6	366.1043	366.1103	1.2	365.1036[M-H]-<br>335.0566[M-H-2CH3]-<br>307.0258[M-H-CH3-C3H5]-	Glycyrol	[76]
17	16.42	C20H16O6	352.0964	352.0947	1.7	353.1021[M+H]+<br>335.0918[M+H-OH]+<br>271.0594[M+H-2CH3-C2H2O]+<br>153.0203[M+H-C13H12O2]+	Semilicoisoflavone B	[79]
18	18.46	C25H24O6	420.1592	420.1573	1.9	419.1501[M-H]-<br>404.1259[-H-CH3]-<br>364.0977[M-H-C4H7]-<br>175.0777[M-H-C14H12O4]-	morusin	[80]

Table S2. The structures and names of chemical ingredients identified from SG.

No.	Name	Structure
1	Rutin	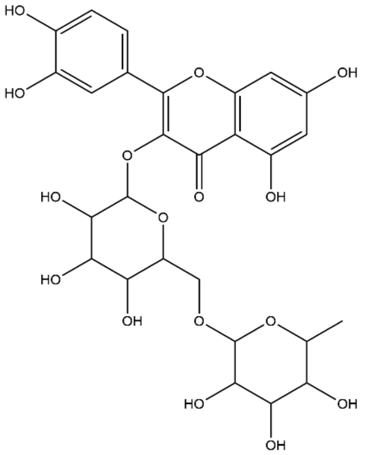
2	Vicenin-2	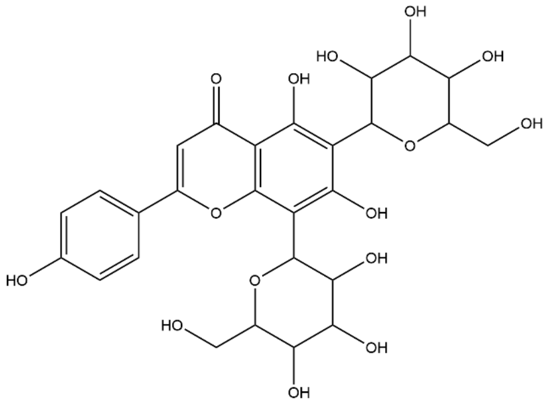
3	Apigenin 7-O-β-D-glucopyranoside	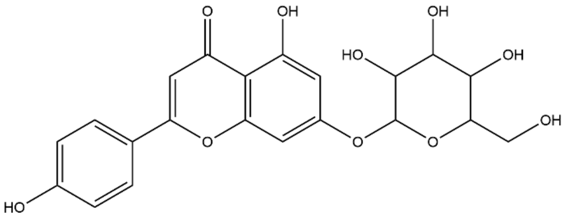
4	Liquiritin apioside	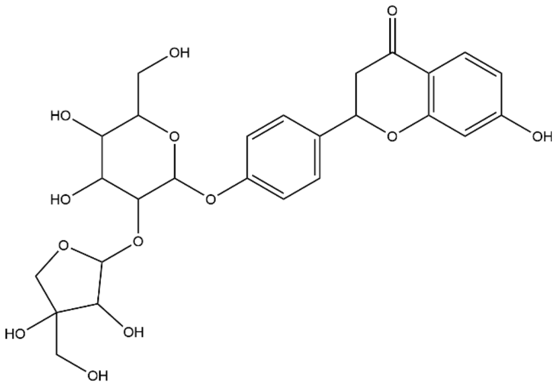
5	Isoliquiritigenin	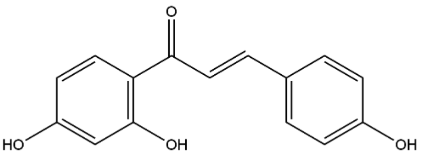

No.	Name	Structure
6	Quercetin	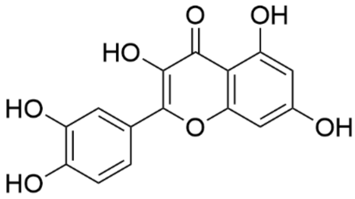
7	Salicylic acid	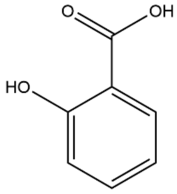
8	Apigenin	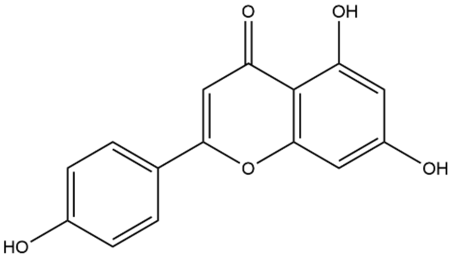
9	Formononetin	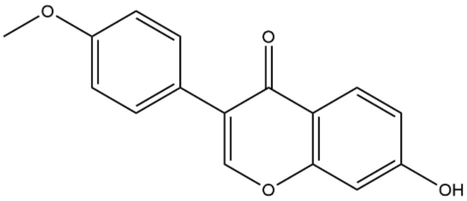
10	Glycyrrhizic acid	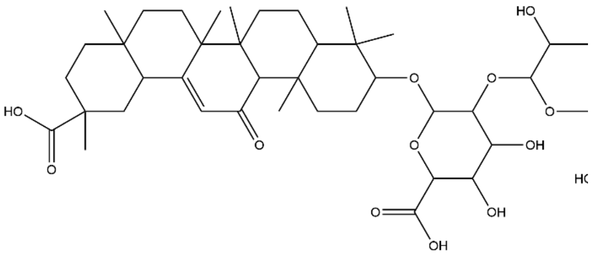
11	Ergosta-6,22-diene-3β,5α,8α-triol	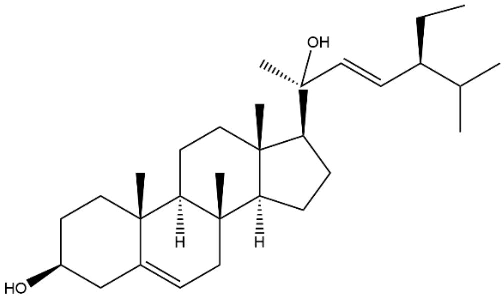
12	Glycycoumarin	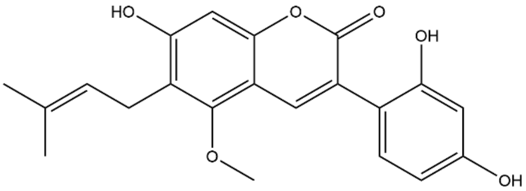

No.	Name	Structure
13	Traxillagenin	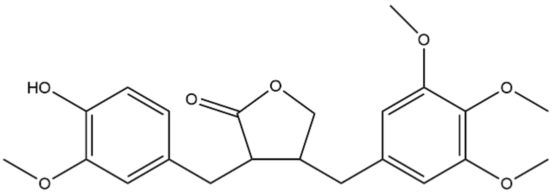
14	Gancaonin L	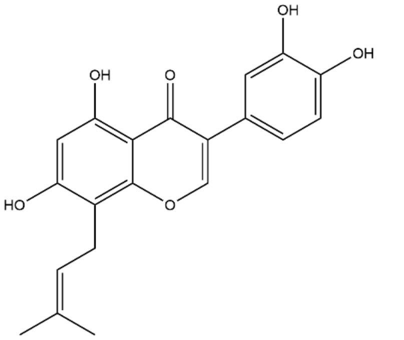
15	Licochalcone A	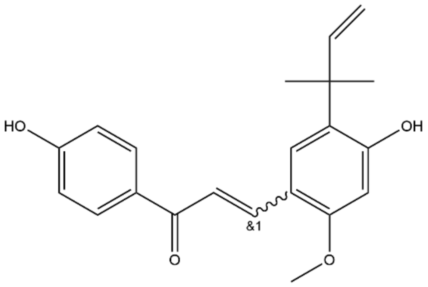
16	Glycyrol	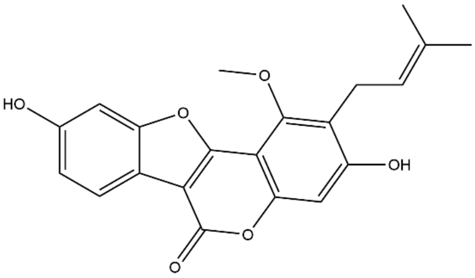
17	Semilicoisoflavone B	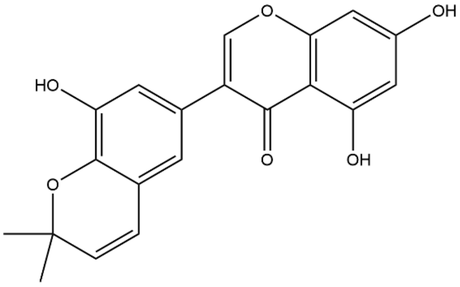
18	Morusin	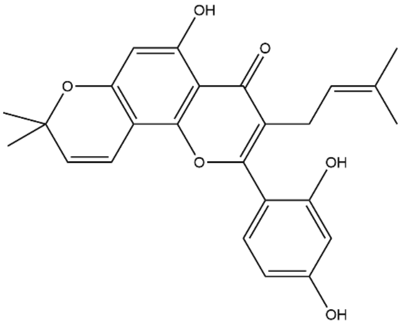

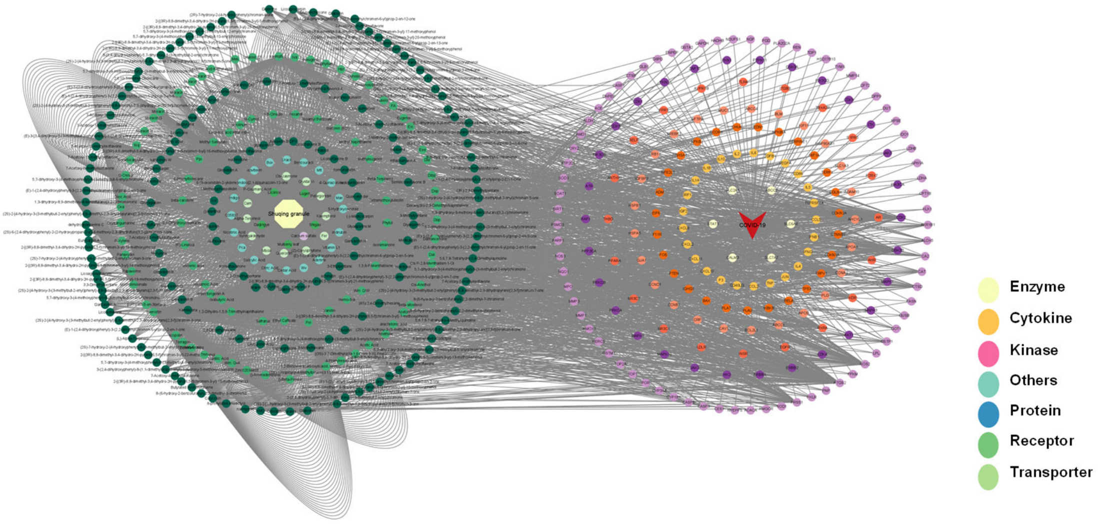

Figure S2. Ingredients of SQ-overlapping targets-COVID-19

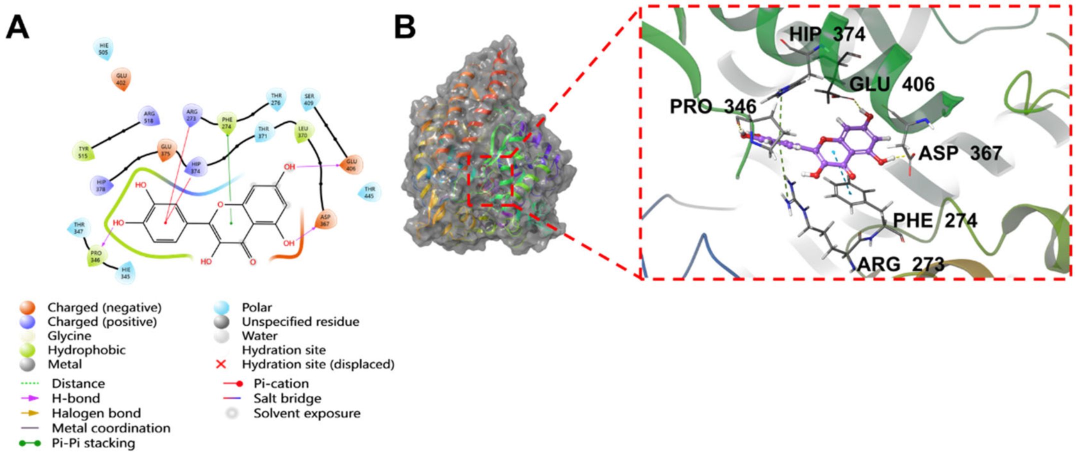

Figure S3. Schematic diagram of the interface between quercetin and ACE2(PDB ID: 1R4L). (A) 2D interaction diagram of quercetin and ACE2. (B) Molecular dock between quercetin and ACE2 crystal structure. Yellow represents hydrogen bonds, blue represents π-π bonds, and green represents π -cation (π Cation) bonds.

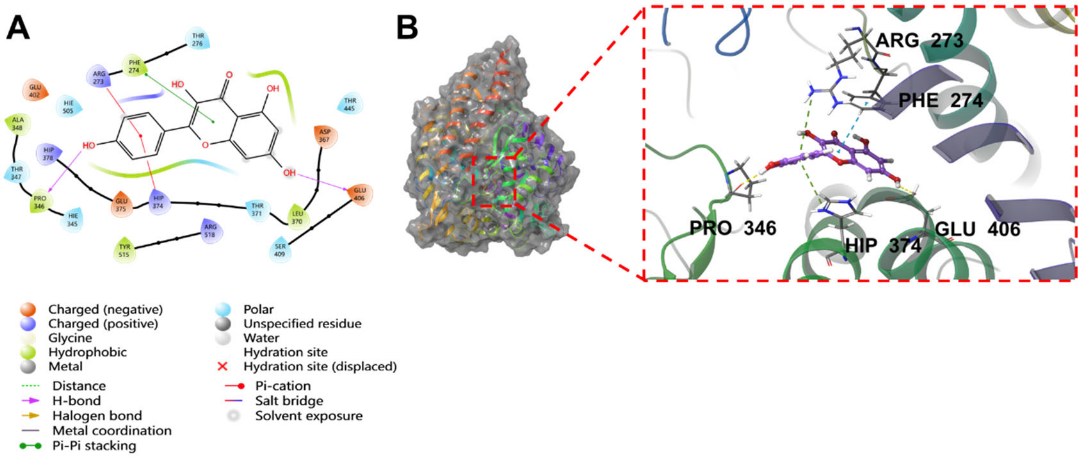

Figure S4. Schematic diagram of the interface between kaempferol and ACE2(PDB ID: 1R4L). (A) 2D interaction diagram of quercetin and ACE2. (B) Molecular dock between quercetin and ACE2 crystal structure. Yellow represents hydrogen bonds, blue represents π-π bonds, and green represents π -cation (π Cation) bonds.

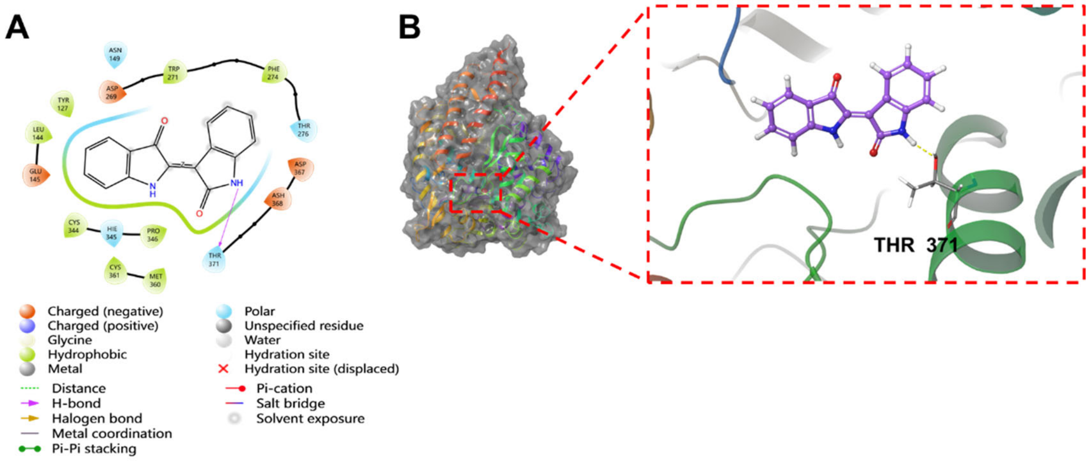

Figure S5. Schematic diagram of the interface between indirubin and ACE2(PDB ID: 1R4L). (A) 2D interaction diagram of indirubin and ACE2. (B) Molecular dock between indirubin and ACE2 crystal structure. Yellow represents hydrogen bonds.

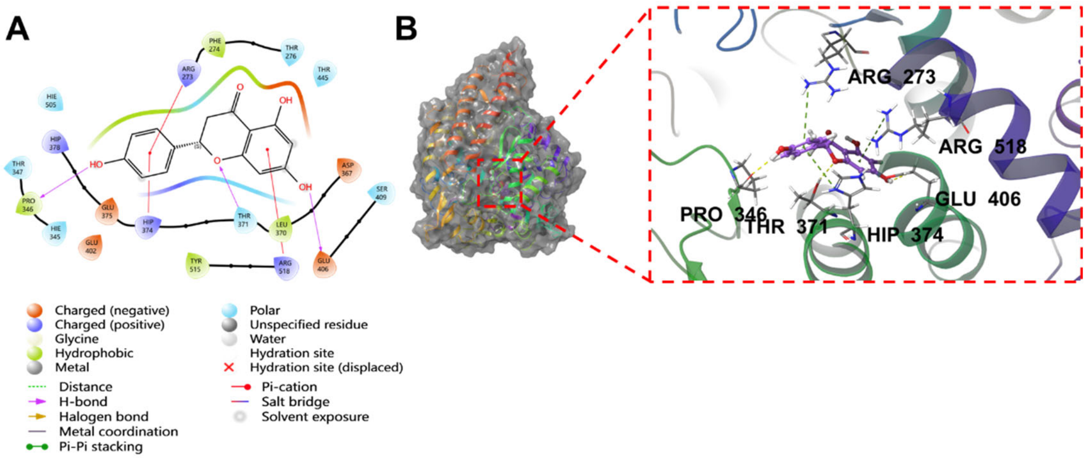

Figure S6. Schematic diagram of the interface between naringenin and ACE2(PDB ID: 1R4L). (A) 2D interaction diagram of naringenin and ACE2. (B) Molecular dock between naringenin and ACE2 crystal structure. Yellow represents hydrogen bonds and green represents π -cation (π Cation) bonds.

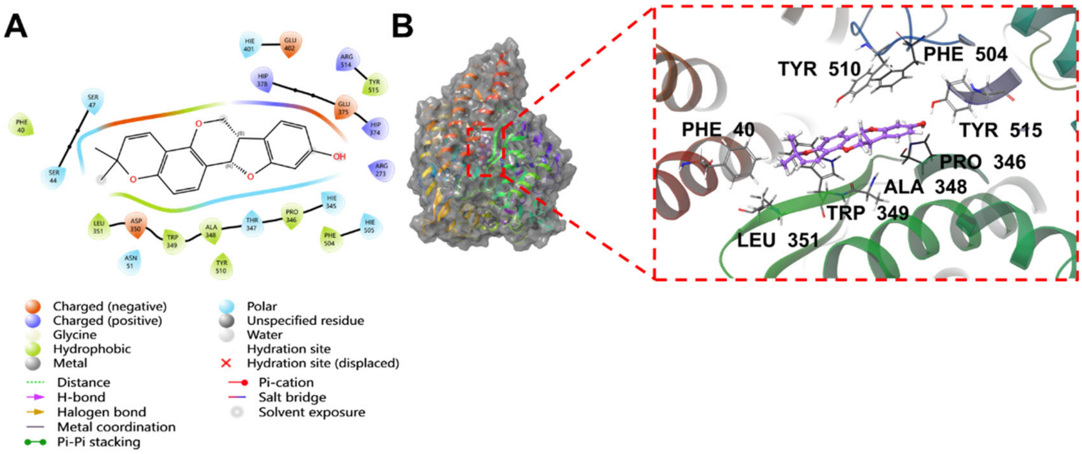

Figure S7. Schematic diagram of the interface between shinpterocarpin and ACE2(PDB ID: 1R4L). (A) 2D interaction diagram of shinpterocarpin and ACE2. (B) Molecular dock between shinpterocarpin and ACE2 crystal structure.

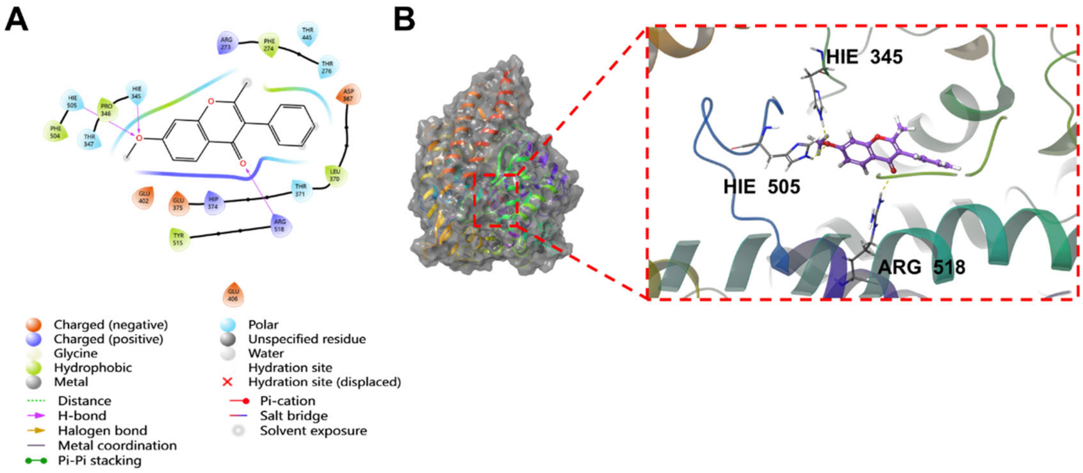

Figure S8. Schematic diagram of the interface between 7-Methoxy-2-methylisoflavone and ACE2(PDB ID: 1R4L). (A) 2D interaction diagram of 7-Methoxy-2-methylisoflavone and ACE2. (B) Molecular dock between 7-Methoxy-2-methylisoflavone and ACE2 crystal structure. Yellow represents hydrogen bonds.

References

69. Zhao Y.; Liu S.; Zhang C.; Liu D.; Zhang T. Analysis on chemical constituents from Glycyrrhizae Radix et Rhizoma by HPLC-Q-TOF-MS (in Chinese). Chin. Tradit. Herb. Drugs. 2016, 12, 2061-2068.

70. He Y.; Tu Z.; Zou A.; Liu H.; Gao L.; Li Y.; Li C. Identification of chemical constituents in Gancao Xiexin Decoction by HPLC-QTOF/MS (in Chinese). Drugs & Clinic. 2021, 11, 2246-2254.

71. Jiang C.; Xiu J.; Cui S.; Xiu H.; Xia Z.; Yang H. Mechanism of Yishen Huashi Granules in treatment of IgA nephropathy based on HPLC-Q-TOF-MS/MS and network pharmacology (in Chinese). Chin. Tradit. Herb. Drugs. 2021, 21, 6576-6585.

72. Li N.; Ren Y.; Zhang C.; Zhong G.; Xiu L.; Liu H.; Chen S.; Chen F.; Li M.; Liao W. Research progress on chemical constituents and pharmacological effects of different varieties of Glycyrrhizae Radix et Rhizoma and predictive analysis of quality markers (in Chinese). Chin. Tradit. Herb. Drugs. 2021, 24, 7680-7692.

73. Zhao P.; Jiao L.; Zhang Z.; Yang X.; Xu J.; Lian Y.; E E. Study of the differences of flavonoids compounds in Glycyrrhiza resi-dues from different countries by UPLC-QTOF-MS (in Chinese). China Food Additives. 2017, 3, 111-116.

74. Zhou Y.; Wang M.; Liao X.; Zhu X.; Peng S.; Ding L. Rapid Identification of Compounds in Glycyrrhiza Uralensis by Liquid Chromatography/Tandem Mass Spectrometry (in Chinese). Chin. J. Anal. Chem. 2004, 2, 174-178.

75. Zhao X.; Zhang J.; Ma X.; Wang Z.; Liu A. A review of the chemical composition of different medicinal parts of lotus (in Chinese). Chin. J. Inf. Tradit. Chin. Med. 2012, 1, 106-109.

76. Cai S.; Zhao H.; Jia M.; Zhao X.; Chi Y.; Zhang W.; Wang H.; Di L. Quality evaluation of fried Glycyrrhizae Radix et Rhizoma pieces by HPLC fingerprint and multicomponent quantitative analysis (in Chinese). Chin. J. Chin. Mater. Med. 2021, 1, 118-124.

77. Ji X. Screening of COX-2 Inhibitory Components of RadixTrachelospermum jasminoides and Its Effect on InflammatoryPain and Analgesia Study on the therapeutic effect of epilepsy (in Chinese). Shaanxi University of Chinese Medicine. 2021.

78. Fang S. Bioactive constituents of flavonoids in the herb residues of licorice (in Chinese). Nanjing University of Chinese Medi-cine. 2016.

79. Hua M.; Zhou Q.; Jiang H.; Dai Y.; Shi D.; Wang P.; Zhang L.; Zhou J. Rapid Screening and Identification of Antioxidant Active Components in Glycyrrhiza uralensis Decoction Pieces (in Chinese). China Pharmacy. 2021,2, 176-181.

80. Deng Z.; Liu J.; Xiong S.; Han L. Metabolite Identification of Morusin in Rats (in Chinese). Food and Drug. 2021, 4, 296-300.
